# Circulating microRNAs (miR-126, miR-197, and miR-223) are associated with chronic kidney disease among elderly survivors of the Great East Japan Earthquake

**DOI:** 10.1186/s12882-019-1651-0

**Published:** 2019-12-21

**Authors:** Ryosuke Fujii, Hiroya Yamada, Mirai Yamazaki, Eiji Munetsuna, Yoshitaka Ando, Koji Ohashi, Hiroaki Ishikawa, Haruki Shimoda, Kiyomi Sakata, Akira Ogawa, Seiichiro Kobayashi, Koji Suzuki, the RIAS study group

**Affiliations:** 1 Department of Preventive Medical Sciences, Fujita Health University School of Medical Sciences, 1-98 Dengakugakubo, Kutsukake-cho, Toyoake, 470-1192 Japan; 2 Department of Hygiene, Fujita Health University School of Medicine, 1-98 Dengakugakubo, Kutsukake-cho, Toyoake, 470-1192 Japan; 3 Department of Clinical Biochemistry, Fujita Health University School of Medical Sciences, 1-98 Dengakugakubo, Kutsukake-cho, Toyoake, 470-1192 Japan; 4 Department of Medical Technology, Kagawa Prefectural University of Health Sciences, 281-1 Hara, Mure-cho, Takamatsu, 761-0123 Japan; 5 Department of Biochemistry, Fujita Health University School of Medicine, 1-98 Dengakugakubo, Kutsukake-cho, Toyoake, 470-1192 Japan; 6 Department of Hygiene and Preventive Medicine, Iwate Medical University, 1-1-1 Idaidori, Yahaba-cho, Shiwa-gun, Iwate, 028-3694 Japan; 7 Iwate Medical University, 1-1-1 Idaidori, Yahaba-cho, Shiwa-gun, Iwate, 028-3694 Japan

**Keywords:** Chronic kidney disease, microRNA, Cardiovascular disease, Molecular epidemiology, Population-based study

## Abstract

**Background:**

A recent study has reported that incidence of chronic kidney disease (CKD) is higher in evacuees, but the molecular mechanism still remains unclear. One plausible hypothesis is a change in vascular function following to psychological distress. In order to assess molecular mechanisms underlying this association, we examined whether cardiovascular disease (CVD)-associated miRNAs (miR-126, miR-197, and miR-223) were associated with CKD among Japanese elderly survivors after an earthquake.

**Methods:**

We analyzed 1385 individuals (670 men and 715 women) who participated in a post-disaster health check-up after the Great East Japan Earthquake, which occurred in 2011. The check-up involved collection of information about lifestyle, clinical history, the degree of housing damage, and baseline measurement of the estimated glomerular filtration rate. Expression levels of miRNAs were determined using real-time polymerase chain reaction. Estimated glomerular filtration rate (eGFR) was calculated using sex, age, and serum creatinine. CKD was defined as eGFR < 60 ml/min/1.73m^2^. The multivariable regression analyses were performed to examine the associations between CVD-associated miRNAs and CKD after adjusting potential confounders.

**Results:**

Mean age (standard deviation) of participants with normal kidney function and CKD was 62.7 (10.6) and 71.9 (8.1) years, respectively. Expression levels of these miRNAs in participants with CKD were significantly lower than normal kidney function (all *p* < 0.001). Even after adjusting for lifestyle, clinical profiles, and psychological distress, significant associations between three miRNAs and CKD still remained. A significant linear association between the cumulative score of these miRNAs and CKD was found (*p* = 0.04).

**Conclusions:**

This cross-sectional study suggested that CVD-associated miRNAs were an important factor of CKD in an elderly Japanese population after earthquake. Future studies need to examine this association in longitudinal dataset.

## Background

Chronic kidney disease (CKD) has become a public health issue in Japan, similar to other developed countries around the world [1, 2]. The number of patients with an estimated glomerular filtration rate (eGFR) less than 60 mL/min/1.73 m^2^ in Japan has reached approximately 13 millions [3]. CKD is associated with cardiovascular disease (CVD) as a major complication [4]. Conversely, CVD is also a risk factor for CKD, which may be mediated by the development of atherosclerosis with vascular calcification and endothelium dysfunction [5].

MicroRNAs (miRNAs), small non-coding RNAs with a length of 18–25 nucleotides, are present as many forms in blood. The biological function of miRNAs is to bind to the 3′-untranslated region of the target mRNAs and thus regulate gene expression [6, 7]. miRNAs have been investigated in various pathophysiological conditions [8, 9] and diseases, including cancer [10–14], CVD [15, 16], diabetes [17], liver disease [18, 19], kidney disease [20], and autoimmune disease [21, 22]. In particular, numerous clinical studies have investigated the associations between CKD and miRNAs in patients and experimental animals [23–28].

A recent prospective study has reported the incidence rate of CKD was higher in evacuees after the Great East Japan Earthquake, a large earthquake occurred in Japan [29]. They speculated that psychological distress after disaster can induce metabolic disorder and hypertension, which lead to kidney dysfunction. However, possible biological mechanisms underlying this association still remain unclear.

Therefore, we focused on vascular function following to psychological distress in the development of CKD, and hypothesized that alterations in levels of vascular function-associated miRNAs [30, 31] may be useful biomarkers for CKD among survivors. Here we investigated whether three circulating CVD-associated miRNAs (miR-126, miR-197, and miR-223) were associated with CKD among elderly survivors of the Great East Japan Earthquake.

## Methods

### Study subjects

The present study was nested within the Research Project for Prospective Investigation of Health Problems Among Survivors of the Great East Japan Earthquake and Tsunami Disaster (RIAS), a longitudinal study performed in the devastated areas in Japan. The RIAS study has been described previously [32]. The primary purpose was to screen the physical and psychological status of the residents in the devastated area after the Great East Japan Earthquake, which occurred in 2011, and to determine long-term effects of the disaster on individual health conditions among adults (over 18 years old) [33–35]. The survey was conducted between September 2011 and February 2012, approximately 6 months after the disaster, in four municipalities (Yamada Town, Otsuchi Town, Kamaishi City and Rikuzentakata City), which are areas that were heavily damaged by the earthquake and tsunami, and in Iwate Prefecture, which is located in the northern part of Japan. We sent out notifications to request a health check examination and a questionnaire to residents in Yamada Town, Otsuchi Town, and Rikuzentakata City (*n* = 12,772, 11,411, and 18,648, respectively). Kamaishi City was excluded in this study because we sent out notifications to residents of temporary housing. In Otsuchi Town, 2172 residents underwent the health examination (participation rate, 19.3%). Of these residents, a total of 2085 individuals participated in the RIAS study (acceptance rate, 96.0%). Among participants in Otsuchi Town (*n* = 2055), 670 participants were excluded for the following reasons: those aged 40 years or younger (*n* = 212); missing value or indeterminable response in the items assessing the degree of housing damage, relocation experience, living environment, psychological factors, smoking status, and habitual alcohol consumption (*n* = 194); past clinical history of cancer, CVD (myocardial infarction, angina, or stroke), kidney disease, or artificial dialysis (*n* = 244); and those with unsuccessful miRNA measurement (*n* = 20). Consequently, a total of 1385 residents (495 men and 890 women) were included in our analysis. Written informed consent was obtained from all participants of this study. This study was approved by the Institutional Review Board of Iwate Medical University (No. H23–69) and the Ethics Review Committee at Fujita Health University (No. HM16–404).

### Definition of CKD

Fasting serum samples were collected from all participants, and centrifuged within an hour of sampling. Biochemical assays were performed using an automated analyzer. eGFR was calculated according to the equation proposed by the Japanese Society of Nephrology: eGFR = 194 × serum creatinine^–1.094^ × age^–0.287^ (× 0.739 for women) [36]. This equation was normally used in epidemiological studies. Individuals with eGFR < 60 mL/min/1.73 m^2^ (CKD stage 3–5) were defined as having CKD.

### Measurement of microRNAs

Quantitative real-time polymerase chain reaction (qRT-PCR) was used to detect expression levels of three circulating CVD-associated miRNAs (miR-126, miR-197, and miR-223) in sera; this procedure has been described in detail elsewhere [18, 37]. Serum miRNAs were isolated using TRIzol reagent (Invitrogen, USA) according to the manufacturer’s instructions. qRT-PCR was performed using the ABI PRISM 7900 Sequence Detection System (Applied Biosystems, Foster City, CA). Relative expression of miRNAs was calculated using the comparative cycle threshold (CT) method (2^−ΔΔCT^). The RNA samples were spiked with *C. elegans* miR-39 (cel-mir-39) as an external control to check extraction of RNA and the efficacy of cDNA synthesis. This is a widely used and practical method for the measurement of circulating miRNAs [38, 39].

### Covariates

Blood pressure was measured twice on both arms using an automatic sphygmomanometer after 5 min of rest. The average value of two readings was calculated and used in our analysis. Anthropometric measurements of height and weight were performed to calculate the body mass index (BMI, kg/m^2^) during the health check-up. Fasting blood glucose levels were determined using an automated analyzer. We collected participants’ information on demographic characteristics (age and sex), lifestyle (smoking status and alcohol consumption), housing environment after the earthquake and tsunami (relocation frequency, degree of housing damage, and current housing environment), and psychological condition (Kessler’s scale) from a self-administered questionnaire. Details of these items in our questionnaire are shown in Additional file 1: Table S1.

### Statistical analysis

The Student’s t-test was used to compare three miRNA expression levels between the group with normal kidney function and the CKD group. In our regression analysis, all participants were divided into tertiles (low, middle, and high) according to the expression levels of each circulating miRNA. The multivariable logistic regression model was used to estimate the odds ratios (ORs) and 95% confidence intervals (CIs) for CKD using the lowest tertile as a reference group. To examine the cumulative effect of these miRNAs on CKD, a miRNA score was calculated by summing the number of the highest tertile of each miRNA; the range was 0 to 3. We then performed logistic regression analysis to estimate the ORs and 95% CIs for the risk of CKD associated with miRNA score increments. These regression analyses were performed after adjusting for covariates, such as age, sex, blood glucose, BMI, systolic blood pressure, smoking status, alcohol consumption, relocation frequency, degree of housing damage, current housing environment, and psychological condition. Statistical significance was defined as a *p*-value less than 0.05, and all tests were two-sided. All statistical analyses were performed using R version 3.5.1 statistical software (R Foundation, Vienna, Austria).

## Results

### Characteristics of participants

Table 1 shows the basic characteristics of participants stratified by CKD status. Mean ages (standard deviations) of participants with normal kidney function and CKD were 62.7 (10.6) and 71.9 (8.1) years, respectively. The values of BMI, systolic blood pressure, blood glucose, and high-density lipoprotein-cholesterol in participants with normal kidney function were significantly different from those values in the CKD group (*p* = 0.02, 0.01, 0.05, and 0.008, respectively).

**Table 1:** Basic characteristics of study subjects according to kidney function

	normal kidney function (*n* = 1156)	CKD (*n* = 229)
Age (year)	62.7 (10.6)	71.9 (8.1)
Male (n, %)	402 (34.8%)	93 (40.6%)
Body mass index (kg/m^2^)	24.1 (3.5)	24.6 (3.1)
Systolic blood pressure (mmHg)	130.4 (19.2)	134.0 (19.7)
Diastolic blood pressure (mmHg)	76.8 (11.2)	76.1 (10.7)
Blood glucose (mmol/l)	6.28 (2.19)	6.58 (2.02)
Triglycerides (mmol/l)^a^	1.33 [0.96, 1.85]	1.45 [0.96, 1.95]
HDL-cholesterol (mmol/l)	1.69 (0.21)	1.60 (0.44)
eGFR (mL/min per 1.73m^2^)	79.8 (13.7)	52.1 (7.0)
log(miR-126)^b^	5.04 (1.04)	4.73 (1.05)
log(miR-197)^b^	2.18 (1.01)	1.81 (0.94)
log(miR-223)^b^	6.17 (1.20)	5.71 (1.08)
	n (%)	n (%)
Smoking status		
Never	828 (71.6%)	180 (78.6%)
Ever	128 (11.1%)	27 (11.8%)
Current	200 (17.3%)	22 (9.6%)
Alcohol consumption		
Never	762 (65.9%)	158 (69.0%)
Ever	160 (13.8%)	28 (12.2%)
Current	234 (20.2%)	22 (9.6%)
Relocation experience		
None	427 (36.9%)	86 (37.6%)
> 1 time	729 (63.1%)	143 (62.4%)
Degree of housing damage		
None	426 (36.9%)	86 (37.6%)
> Partially destroyed	730 (63.1%)	143 (62.4%)
Living in a shelter		
Yes	512 (44.3%)	95 (41.5%)
Psychological distress (K6 > 13)		
Yes	75 (6.5%)	12 (5.2%)

*CKD* Chronic kidney disease, *eGFR* Estimated glomerular filtration rate

^a^ Expressed as median and interquartile range

^b^ Expressed as arbitrary scale

### Associations between three circulating miRNAs and CKD

**Fig. 1:**
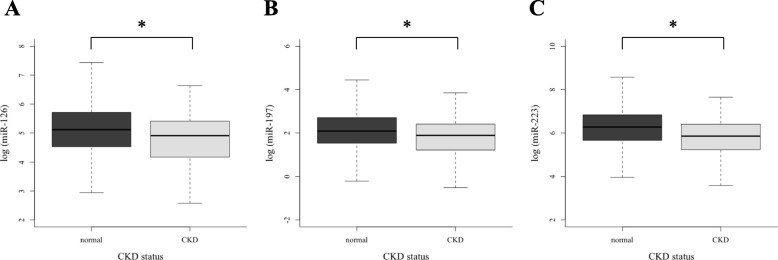
The comparison of three circulating CVD-associated micro RNAs (miR-126, miR-197, and miR-223) between normal and CKD group

The associations between three circulating miRNAs (miR-126, miR-197, and miR-223) and CKD status are shown in Fig. 1. Expression levels of all three miRNAs were significantly lower in the CKD group compared with the normal kidney function group (all *p* < 0.001). Table 2 shows crude and adjusted ORs with 95% CIs for the risk of CKD according to different tertiles of each miRNA. Individuals in the highest tertile of each miRNA had a significantly lower risk for CKD compared with the lowest tertile of the miRNA (OR [95% CI] = 0.67 [0.45–0.98], 0.67 [0.46–0.99], and 0.53 [0.35–0.79], for miR-126, miR-197, and miR-223, respectively).

**Table 2:** Associations between tertiles of three microRNAs and chronic kidney disease (eGFR < 60)

			Crude model		Fully adjusted model	
microRNA		CKD (%)	OR (95%CI)	*p*	OR (95%CI)	*p*
miR-126	Low (*n* = 457)	98 (21.4%)	Reference		Reference	
	Middle (*n* = 471)	76 (16.1%)	0.71 (0.51–0.98)	0.04	0.74 (0.52–1.06)	0.11
	High (*n* = 457)	55 (12.0%)	0.50 (0.35–0.72)	< 0.001	0.67 (0.45–0.98)	0.04
miR-197	Low (*n* = 457)	100 (21.9%)	Reference		Reference	
	Middle (*n* = 471)	75 (15.9%)	0.68 (0.48–0.94)	0.02	0.72 (0.50–1.02)	0.07
	High (*n* = 457)	54 (11.8%)	0.48 (0.33–0.68)	< 0.001	0.67 (0.46–0.99)	0.05
miR-223	Low (*n* = 457)	110 (24.1%)	Reference		Reference	
	Middle (*n* = 471)	75 (15.9%)	0.59 (0.43–0.83)	0.002	0.69 (0.49–0.98)	0.04
	High (*n* = 457)	44 (9.6%)	0.34 (0.23–0.49)	< 0.001	0.53 (0.35–0.79)	0.002

*eGFR* Estimated glomerular filtration rate, *CKD* Chronic kidney disease, *OR* Odds ratio, *95% CI* 95% confidential interval

Adjustment for sex, age, blood glucose levels, systolic blood pressure, body mass index, smoking status, alcohol consumption, psychological distress, relocating experience, degree of housing damage, and living in a shelter

### Associations between miRNA score and CKD

Table 3 shows adjusted ORs with 95% CIs for the risk of CKD according to different miRNA scores. The logistic regression model indicated that participants with the highest score (score: 3), which integrated the three miRNAs, had significantly lower ORs for CKD (eGFR < 60) compared with the lowest score (score: 0) (OR [95% CI] = 0.46 [0.26–0.79]), but no significant risk reduction in other scores (score: 1 and 2) was found (*p* = 0.92 and 0.83). An additional analysis confirmed the significant linearity in ORs for CKD according to the miRNA score (*p* = 0.04).

**Table 3:** Association between cumulative score of three miRNAs and chronic kidney disease (eGFR < 60)

microRNA score (n)	OR (95%CI)	*p*	*trend p*
0 (*n* = 715)	Reference		0.04
1 (*n* = 216)	1.02 (0.66–1.56)	0.19	
2 (*n* = 207)	1.05 (0.66–1.63)	0.92	
3 (*n* = 247)	0.46 (0.26–0.79)	0.007	

*eGFR* Estimated glomerular filtration rate, *OR* Odds ratio, *95% CI* 95% confidential interval

Adjustment for sex, age, blood glucose levels, systolic blood pressure, body mass index, smoking status, alcohol consumption, psychological distress, relocating experience, degree of housing damage, and living in a shelter

## Discussion

The present study showed that three vascular function-associated miRNAs (miR-126, miR-197, and miR-223) were significantly associated with CKD among survivors of the earthquake. This result suggested that difference in vascular function after the natural disaster might be an important factor of a lower kidney function. Our findings were consistent with the result observed in the case-control study in Belgium [28].

miR-126 is one of the most abundantly expressed miRNAs in endothelial cells (ECs), and is a well-studied miRNA in vascular biology and diseases [40, 41]. Fish et al. suggested that endogenous miR-126 may be associated with various vascular functions (angiogenesis, leukocyte adhesion, and inflammation) in atherosclerotic lesions by down-regulating expression of proteins involved in signaling pathways such as Sprouty-related enabled/VASP homology domain-containing protein 1 and phosphatidyl inositol 3-kinase regulatory beta [42]. In their discussion, decreases in these molecules activate rapidly accelerated fibrosarcoma in the mitogen-activated protein kinase signaling pathway, thus increasing expression of vascular endothelial growth factor and promoting angiogenesis. In addition to angiogenesis, Harris et al. found that increasing miR-126 levels in ECs inhibits vascular cell adhesion molecule 1 expression, resulting in decreased leukocyte adhesion to ECs [43]. Moreover, miR-126 increases sirtuin1 and superoxide dismutase-2 expression, thus decreasing oxidative stress in ECs [44]. Accordingly, higher levels of circulating miR-126 may be correlated with maintenance of vascular function and a reduced risk of CKD.

Our results showed a negative association between miR-223 and eGFR. miR-223 is originally identified as an important regulator of hematopoietic system and is associated with various biological function and development of cancer, inflammatory diseases, and diabetes [45]. A recent paper using rats suggested that inhibition of miR-223 in injured carotid could be a therapeutic method to prevent vascular calcification [46]. On the other hand, another experimental study demonstrated that serum miR-223 is lower in ApoE knock-out mice, which show vascular calcification, than in wild-type counterparts [47]. This study also showed that expression levels of miR-223 in serum were opposite of the levels in tissue samples. As shown in the miRDB (http://mirdb.org/), miR-223 targets insulin-like growth factor 1 receptor (IGF-1R), and overexpression of miR-223 in plaque vascular smooth muscle cells (VSMCs) induces a decrease in IGF-1R [48]. Many researchers have demonstrated that increased expression of IGF-1R in VSMCs prevents cell proliferation and migration, a crucial and primary pathophysiological process in the atherosclerotic cascade [48, 49]. Considering these previous studies, decreased expression levels of circulating miR-223 may be associated with increased expression of miR-223 in VSMCs, leading to protective effects against the atherosclerotic process.

miR-197 has been extensively studied in the field of cancer biology, including aspects such as apoptosis, proliferation, and metastasis [50–52]. We found that higher levels of circulating miR-197 were significantly associated with better kidney function. This result must be interpreted cautiously because other previous studies have shown a different relationship between miR-197 and CVD [30, 31]. Even though they did not provide clear explanations, one plausible mechanism is that miR-197, which is highly expressed in platelets, activates platelets and plays a role in the development of CVD [53]. However, the function of this miRNA in platelets is not fully understood. Further studies are needed to elucidate the function of miR-197 in human platelets, which can lead to biological mechanisms underlying the true relationship between miR-197 and CKD.

The major finding in this study is the significant association between three circulating CVD-associated miRNAs and CKD among survivors after the earthquake. However, the present study also has some limitations. First, the design of this study was cross-sectional, which does not allow us to assess a possible causal relationship between CVD-associated miRNAs and CKD. Further studies with a longitudinal dataset may allow examination of a causal relationship between CVD-associated miRNAs and CKD. Second, our subjects were homogeneous in terms of ethnicity, age, and regional features. Accordingly, these results must be confirmed in other populations with different ethnicities, environmental factors, dietary habits, and lifestyles.

## Conclusion

In summary, this study may provide a novel insight that CVD-associated circulating miRNAs were significantly associated with eGFR among survivors after the earthquake and tsunami disaster. These associations indicate that difference of CVD-associated miRNAs can contribute to a lower kidney function following to natural disaster. Further longitudinal studies in other populations are expected to assess this association among adults after natural disaster.

## Supplementary information

**Additional file 1: Table S1.** Details of the self-administered questionnaire used in this study.

## Data Availability

The datasets used and/or analyzed during the current study are available from the corresponding author on reasonable request.

## Abbreviations

CI: Confidential interval
CKD: Chronic kidney disease
CVD: Cardiovascular disease
EC: Endothelial cell
eGFR: Estimated glomerular filtration rate
IGF-1R: Insulin-like growth factor-1 receptor
miRNA: microRNA
OR: Odds ratio
PCR: Polymerase chain reaction
RIAS: Research Project for Prospective Investigation of Health Problems Among Survivors of the Great East Japan Earthquake and Tsunami Disaster
VSMC: Vascular smooth muscle cell
