# Supplementary material for: Circulating microRNAs (miR-126, miR-197, and miR-223) are associated with chronic kidney disease among elderly survivors of the Great East Japan Earthquake
Source: BMC Nephrol. 2019 Dec 21;20:474. doi: 10.1186/s12882-019-1651-0 (PMC6925484; doi:10.1186/s12882-019-1651-0)
Supplement: Supplementary file 1 — Additional file 1: Table S1. Details of the self-administered questionnaire used in this study. [file 12882_2019_1651_MOESM1_ESM.pdf]

**Circulating cardiovascular disease-associated microRNAs are associated with chronic kidney disease in elderly survivors of the Great East Japan Earthquake.**

Ryosuke Fujii, Hiroya Yamada, Eiji Munetsuna, Mirai Yamazaki, Yoshitaka Ando, Koji Ohashi, Hiroaki Ishikawa, Haruki Shimoda, Kiyomi Sakata, Akira Ogawa, Seiichiro Kobayashi, Koji Suzuki, the RIAS study

**Supplemental Table 1. Details of the self-administered questionnaire used in this study.**

| Items | Questions | Selection branch | Code for our analyses (n) |
|---|---|---|---|
| Smoking status | What is your smoking status? | 1) never<br>2) ever<br>3) current | 0) ever or never<br>1) current |
| Alcohol consumption | What is your status for alcohol consumption? | 1) never<br>2) ever<br>3) current | 0) ever or never<br>1) current |

| | | | |
|---|---|---|---|
| Relocating experience | How many times have you moved after the disaster? | 1) none<br>2) once<br>3) twice<br>4) 3 times<br>5) ≥ 4 times | 0) none<br>1) ≥ once |
| Degree of housing damage | (This item was assessed by dataset of the extent of damage for each administrative district with seven categories) | 1) housing destroyed<br>2) flooded<br>3) partially housing destroyed<br>4) partially flooded<br>5) housing destroyed and partially flooded<br>6) partially destroyed and flooded<br>7) not flooded | 0) no damage versus<br>1) ≥ partially destroyed |
| Current housing environment | Which residence do you mainly live in now? | 1) own home<br>2) home of relative or acquaintance<br>3) evacuation center<br>4) prefabricated temporary housing<br>5) rented or new accommodation<br>6) other residence | 0) others<br>1) living in a shelter |
| Kessler's scale (K6) | How often an individual has felt the following six items (nervous, hopeless, restless, or fidgety, so sad that nothing cheer them up, everything requires effort, and worthless) in the month? | 5-point Likert scale | 0) < 13<br>1) ≥ 13 |
